# NEPHROLOGY CARE AND MORTALITY RATES AMONG PATIENTS WITH END-STAGE RENAL DISEASE IN PUERTO RICO AND THE UNITED STATES

**DOI:** 10.1093/geroni/igz038.2083

**Published:** 2019-11-08

**Authors:** Maricruz Rivera-Hernandez, Shailender Swaminathan, Rebecca Thorsness, Yoojin Lee, Rajnish Mehrotra, Benjamin Sommers, Amal N Trivedi

**Affiliations:** 1 Brown University, Providence, Rhode Island, United States; 2 University of Washington, Seattle, Washington, United States; 3 Harvard, Boston, Massachusetts, United States

## Abstract

Hispanics with incidence of end-stage renal disease (ESRD) have shown lower mortality despites their high incidence rates; However, prior research has excluded Puerto Rico (PR). This study compared mortality rates and predialysis nephrology care among Hispanics in the US, Hispanics in PR, and Whites in the US with ESRD from 2006-2015. We identified 791,443 patients using the Renal Management Information System. The primary outcome was age-adjusted 1-year mortality beginning with the 91st day following dialysis initiation. Secondary outcomes were the presence of arteriovenous fistula or graft at dialysis initiation, and receipt of predialysis nephrology care. Despite higher rates of insurance coverage, we identified substantial disparities in access to recommended nephrology care between PR and the US. In addition, the adjusted absolute difference in mortality rates was higher for PR Hispanics. This finding indicates shortcomings in quality of care for Puerto Rico with serious chronic illness and complex care needs.

